# Bloodstream infections due to Carbapenem-Resistant Enterobacteriaceae in hematological patients: assessment of risk factors for mortality and treatment options

**DOI:** 10.1186/s12941-023-00586-y

**Published:** 2023-05-18

**Authors:** Lining Zhang, Sisi Zhen, Yuyan Shen, Tingting Zhang, Jieru Wang, Jia Li, Qingsong Lin, Zhijian Xiao, Yizhou Zheng, Erlie Jiang, Mingzhe Han, Jianxiang Wang, Sizhou Feng

**Affiliations:** Hematopoietic Stem Cell Transplantation Center, State Key Laboratory of Experimental Hematology, Haihe Laboratory of Cell Ecosystem, Institute of Hematology & Blood Diseases Hospital, National Clinical Research Center for Blood Diseases, Chinese Academy of Medical Sciences & Peking Union Medical College, Tianjin, 300020 China

**Keywords:** Carbapenem-resistant Enterobacteriaceae, Bloodstream infection, Hematological patient, Carbapenemase gene, Antimicrobial regimen

## Abstract

**Purpose:**

Bloodstream infection (BSI) caused by Carbapenem-Resistant Enterobacteriaceae (CRE) are associated with poor outcomes in hematological patients. The aim of this study was to identify risk factors for mortality and evaluate the value of epidemiological feature of carbapenemases in guiding antimicrobial treatment options.

**Methods:**

Hematological patients with monomicrobial CRE BSI between January 2012 and April 2021 were included. The primary outcome was all-cause mortality 30 days after BSI onset.

**Results:**

A total of 94 patients were documented in the study period. *Escherichia coli* was the most common Enterobacteriaceae, followed by *Klebsiella pneumoniae*. 66 CRE strains were tested for carbapenemase genes, and 81.8% (54/66) were positive, including NDM (36/54), KPC (16/54), IMP (1/54). Besides, one *E. coli* isolate was found to express both NDM and OXA-48-like genes. Overall, 28 patients received an antimicrobial treatment containing ceftazidime-avibactam (CAZ-AVI), of which 21 cases were combined with aztreonam. The remaining 66 patients were treated with other active antibiotics (OAAs). The 30-day mortality rate was 28.7% (27/94) for all patients, and was only 7.1% ((2/28) for patients treated with CAZ-AVI. In multivariate analysis, the presence of septic shock at BSI onset (OR 10.526, 95% CI 1.376–76.923) and pulmonary infection (OR 6.289, 95% CI 1.351–29.412) were independently risk factors for 30-day mortality. Comparing different antimicrobial regimens, CAZ-AVI showed a significant survive benefit than OAAs (OR 0.068, 95% CI 0.007–0.651).

**Conclusion:**

CAZ-AVI-containing regimen is superior to OAAs for CRE BSI. As the predominance of *bla*NDM in our center, we recommend the combination with aztreonam when choose CAZ-AVI.

## Introduction

The emergence and global spread of Carbapenem-resistant Enterobacteriaceae (CRE) has represented a major threat to public health. Due to limited treatment options, CRE infections are associated with high morbidity and mortality, especially for CRE bloodstream infection (BSI). Patients with hematologic malignancies, who frequently experience prolonged neutropenia, immunosuppression, chemotherapy-induced mucositis and invasive procedures, are more vulnerable to CRE infection and usually with dismal clinical outcomes. Although many studies on CRE infection have been reported in the literature, there are only a few on patients with hematologic malignancies. Therefore, we conducted a retrospective study to describe the clinical and microbiological outcomes in hematological patients with BSI due to CRE. We aimed to assess risk factors for mortality and evaluate different antibiotic therapies, especially the efficacy of treatment options guided by the prevalence trends of carbapenemases.

## Materials and methods

### Study setting

This retrospective study was conducted at a blood disease hospital with 767-bed in Tianjin, China. Hematological patients with monomicrobial CRE BSI from January 2012 to April 2021 were included in this study. Patients were excluded if they had polymicrobial bacteremia. This study was approved by the ethical committee of the Institute of Hematology and Blood Diseases Hospital, Chinese Academy of Medical Sciences.

### Data collection

The following data were recorded for each patient: sex, age, underlying hematological disease, length of stay, chemotherapy, hematopoietic stem cell transplantation (HSCT), history of previous hospitalizations, neutropenia, mucositis, carriage or infection with multidrug-resistant organism (MDRO) in the previous 3 months. Comorbidities were evaluated by the Charlson comorbidity index (CCI), and the Pitt bacteremia score (PBS) was determined on the day of the index culture to assess the severity of illness. For patients with more than one episode of CRE BSI, only data relevant to the first episode was collected. All patients were followed up until 30 days after the BSI episode. The primary outcome was all-cause mortality 30 days after infection onset. Risk factors were evaluated by comparing the variables of survivors with those of non-survivors.

### Microbiology

Isolate identification and antimicrobial susceptibility test were performed using the Vitek 2 automated system (bioMérieux). Antibiotic susceptibilities were defined according to the guidelines of the Clinical and Laboratory Standards Institute (CLSI) M100. The predominant carbapenemase genes (*bla*KPC, *bla*NDM, *bla*OXA-48, *bla*IMP, *bla*VIM and *bla*OXA-23) was detected by polymerase chain reaction (PCR) as gold standard [1]. Colloidal gold immunochromatography was used for rapid identification of carbapenemase according to manufacturer’s recommendation [2].

### Definitions

CRE are defined as those Enterobacteriaceae that are resistant to any carbapenem antimicrobial (i.e., minimum inhibitory concentrations (MIC) of ≥ 4 mg/l for doripenem, meropenem, or imipenem or ≥ 2 mg/l for ertapenem) or documented to produce carbapenemase [3]. The onset of BSI was considered as the date of collection of the first positive blood culture. Antimicrobial treatment given before susceptibility testing was defined as empirical therapy and treatment administered after the susceptibility testing was defined as definitive therapy. Monotherapy was defined as a regimen including one agent with in vitro activity and combination therapy was defined as treatment with two or more agents with in vitro activity. All antimicrobial agents included in the statistics were used for at least 48 h. Acute leukemia (AL) in complete remission (CR), lymphoma in CR or partial remission, refractory anemia of myelodysplastic syndrome (MDS) and severe aplastic anemia (SAA) untreated were defined as standard risk, while AL in induction failure or relapse, lymphoma in stable disease or progression, MDS/SAA transfusion dependence with no response to treatment were classified as high risk [4, 5]. Neutropenia was defined as an absolute neutrophil count < 0.5 × 10^9^ cells/L.

### Statistical analysis

Data analyses were conducted using the statistical package SPSS 22.0. Univariate analysis was performed by a chi-square test or Fisher’s exact test for categorical variables and Student’s t test for continuous variables. Survival curve was performed using the Kaplan-Meier method. Variables for which the *p*-value was ≤ 0.10 in the univariate analysis were included in a logistic regression model. All *p*-values were based on two tailed statistical analyses and *p*-values less than 0.05 were considered statistically significant.

## Results

### Clinical and microbiological characteristics

Overall, 94 patients with CRE BSI were documented in the study period. The clinical characteristics are described in Table 1. They had a median age of 38 (IQR, 25–49) years, and 58 cases (62%) were male. 70 (74.5%) patients were diagnosed with AL, 13 (13.8%) with SAA, 9 (9.6%) with MDS, 2 (2.1%) with non-Hodgkin’s lymphoma. Among them, 20 (21.3%) patients received allogeneic HSCT. Most of patients (96.8%) were neutropenic when developed CRE BSI, and 72.3% (68/94) had neutropenia with a duration of more than 14 days. 15 (16%) cases presented with septic shock and 34 (36.2%) with pulmonary infection at BSI onset. Bacterial pneumonia was the most common infections (n = 16), 10 cases of which were caused by CRE. Other pathogens were *Acinetobacter baumannii*, *Stenotrophomonas maltophilia* and *Enterococcus faecalis*. Invasive pulmonary fungal infection followed (n = 14), including pulmonary aspergillosis, mucormycosis and candidiasis. In addition, 4 patients were mixed bacterial and fungal pulmonary infection. Other co-infections were gastroenteritis (n = 15), perianal infection (n = 14), oral mucositis (n = 7), skin and soft tissue infection (n = 5), pharyngitis (n = 4). Besides, 56 patients had a history of MDRO colonization or infection in the previous 3 months.

**Table 1:** Clinical characteristics and risk factors for 30-day mortality in patients with CRE BSI

	All Patients (n = 94)	Survivors (n = 67)	Non-survivors (n = 27)	*P* value
**Sex**				0.530
Male	58 (61.7)	40 (69.0)	18 (31.0)	
Female	36 (38.3)	27 (75.0)	9 (25.0)	
**Age (years), median (IQR)**	38 (25–49)	38 (27–49)	33 (18–49)	0.254
**Charlson comorbidity score**				0.205
2	80 (85.1)	59 (73.8)	21 (26.3)	
3–4	14 (14.9)	8 (57.1)	6 (42.9)	
**Underlying hematological disease**				< 0.001
Standard-risk group	36 (38.3)	34 (94.4)	2 (5.6)	
High-risk group	58 (61.7)	33 (56.9)	25 (43.1)	
**Received HSCT**				0.678
Yes	20 (21.3)	15 (75.0)	5 (25.0)	
No	74 (78.7)	52 (70.3)	22 (29.7)	
**CRE isolates**				0.052
*Escherichia coli*	48 (51.1)	35 (72.9)	13 (27.1)	
*Klebsiella pneumoniae*	36 (38.3)	22 (61.1)	14 (38.9)	
Others	10 (10.6)	10 (100)	0 (0)	
**MDRO carriage/infection**				0.968
Yes	56 (59.6)	40 (71.4)	16 (28.6)	
No	38 (40.4)	27 (71.1)	11 (28.9)	
**Meropenem MICs (mg/L)**				0.806
> 8	78 (83.0)	11 (68.8)	5 (31.2)	
≤ 8	16 (17.0)	56 (71.8)	22 (28.2)	
**Length of neutropenia**				0.012
≥ 14 days	68 (76.4)	46 (67.6)	22 (32.4)	
< 14 days	21 (23.6)	20 (95.2)	1 (4.8)	
**Mucositis**				0.188
Yes	29 (30.9)	18 (62.1)	11 (37.9)	
No	65 (69.1)	49 (75.4)	16 (24.6)	
**Pitt bacteremia score**				0.002
≥ 2	31 (33.3)	16 (51.6)	15 (48.4)	
< 2	62 (66.7)	51 (82.3)	11 (17.7)	
**Septic shock**				< 0.001
Yes	15 (16)	4 (26.7)	11 (73.3)	
No	79 (84)	63 (79.7)	16 (20.3)	
**Co-infections**				
Pulmonary infection	34 (36.2)	17 (25.4)	17 (63.0)	0.001
Gastroenteritis	15 (16.0)	10 (14.9)	5 (18.5)	0.667
Perianal infection	14 (14.9)	12 (17.9)	2 (7.4)	0.196
others	16 (17.0)	9 (13.4)	7 (25.9)	0.145

IQR, interquartile range; HSCT, hematopoietic stem cell transplantation; MDRO, multidrug-resistant organism

**Fig. 1:**
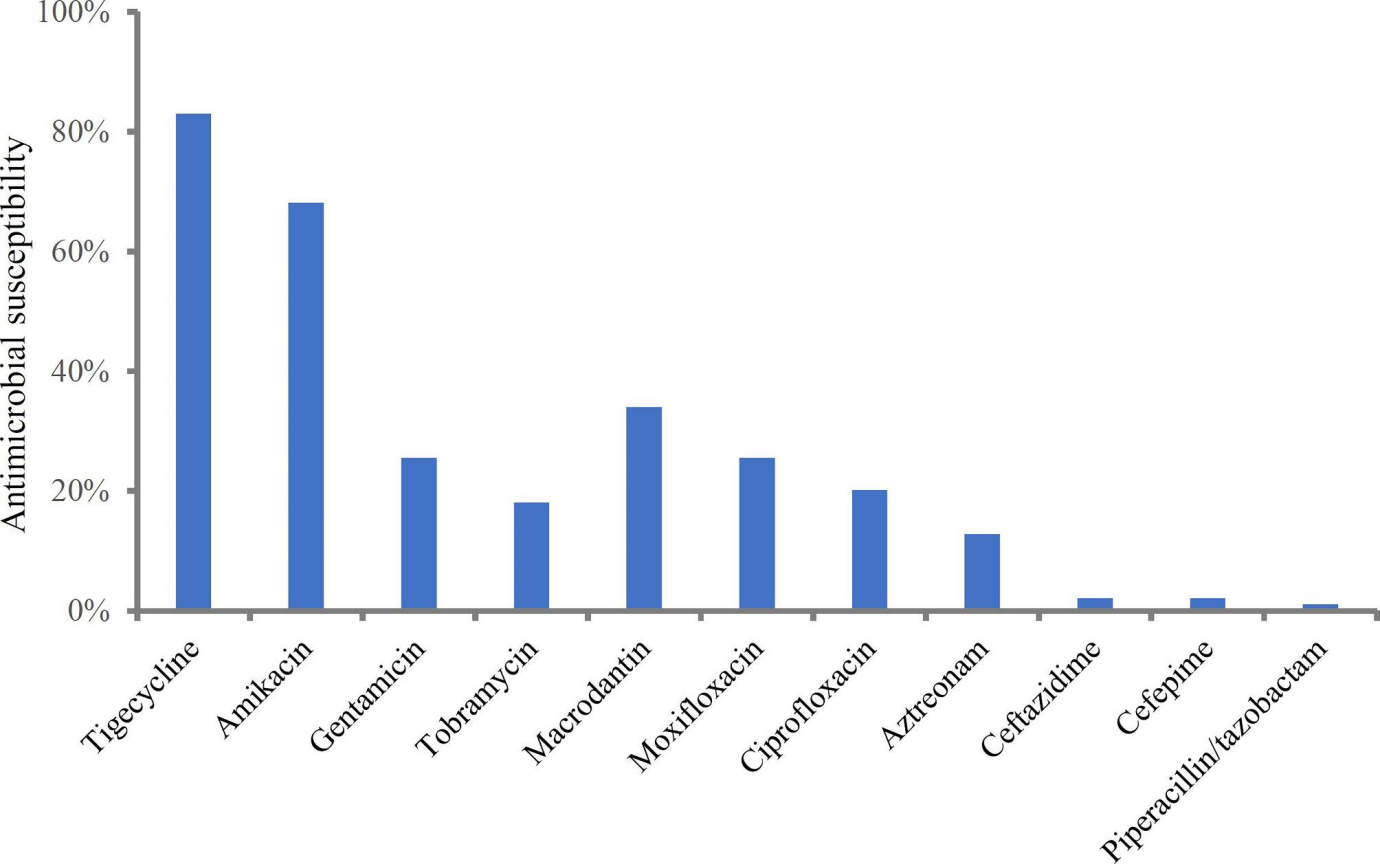
Distribution of antimicrobial susceptibility of CRE isolates

Of the 94 CRE isolates, *Escherichia coli* was the most common Enterobacteriaceae (n = 48), followed by *Klebsiella pneumoniae* (n = 36), *Enterobacter cloacae* (n = 8), *Enterobacter aerogenes* (n = 1) and *Raoultella planticola* (n = 1). The antimicrobial susceptibilities of these isolates are described in Fig. 1. Of note, the majority (83%) of CRE isolates were susceptible to tigecycline, over half (68.1%) were susceptible to amikacin. Other classes of antimicrobials, such as fluoroquinolones, cephalosporins, piperacillin/tazobactam, exhibited high levels of resistance. Meropenem MICs were > 8 mg/L for 78 (83.0%) isolates, and ≤ 8 mg/L for 16 (17.0%) isolates.

**Fig. 2:**
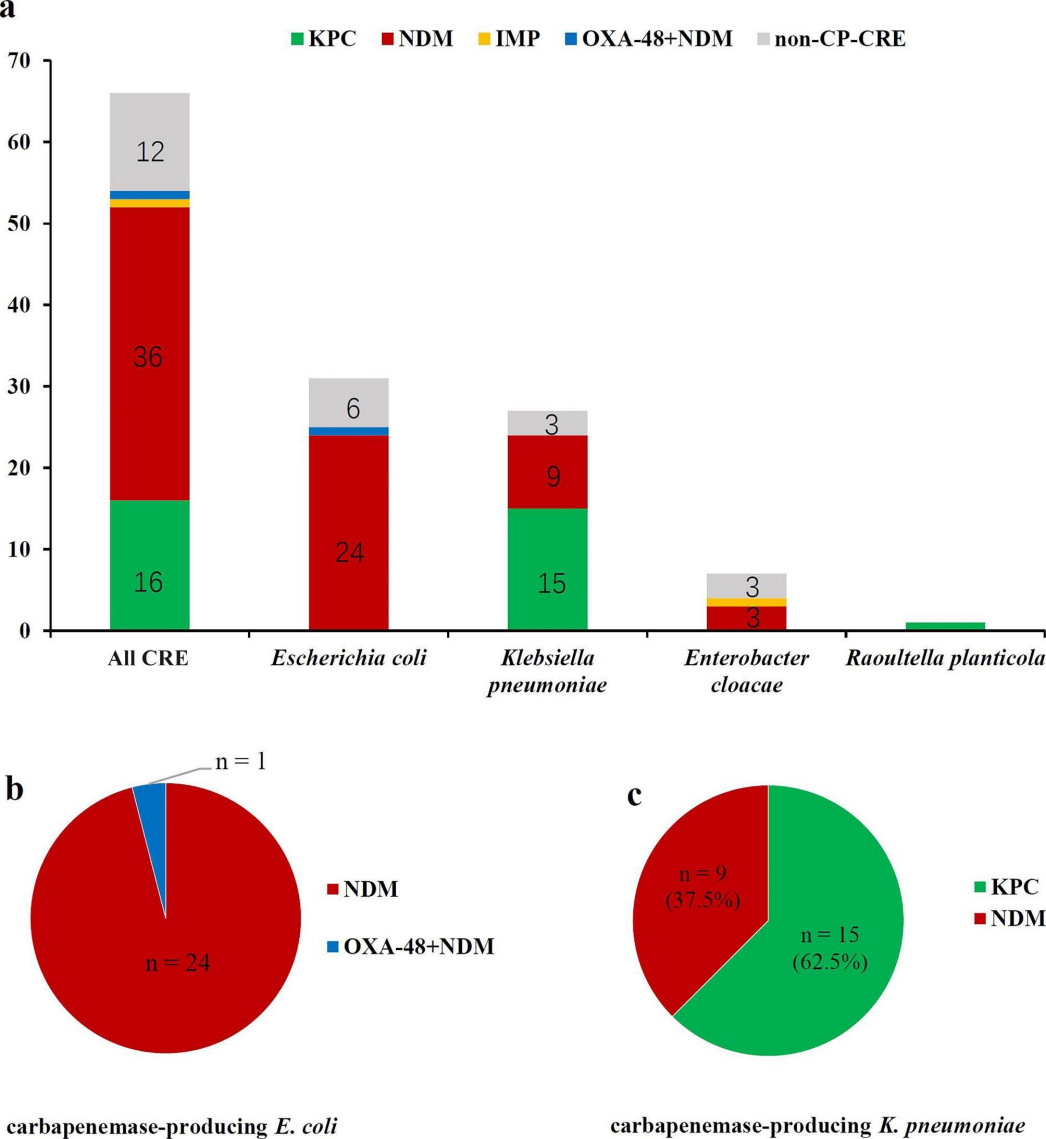
Distribution of carbapenemase genes by PCR in 66 CRE strains **(a)** Distribution of carbapenemase genes in 25 carbapenemase-producing *E. coli***(b)** and 24 carbapenemase-producing *K. pneumoniae***(c)**

A total of 66 CRE strains were detected for the presence of genes encoding carbapenemase by PCR and colloidal gold immunochromatography simultaneously. As shown in Fig. 2, carbapenemase genes were positive in 81.8% (54/66) of CRE isolates, including NDM (66.7%, 36/54), KPC (29.6%, 16/54), IMP (1.9%, 1/54). Besides, one *E. coli* isolate was found to express both NDM and OXA-48-like genes. No strain was positive for VIM or OXA-23. Interestingly, all 25 carbapenemase-producing *E. coli* expressed NDM genes. Among 24 carbapenemase-producing *K. pneumoniae*, the most common carbapenemase gene was KPC (n = 15), followed by NDM (n = 9). Among 4 carbapenemase-producing *E. cloacae*, 3 strains were KPC-producers, 1 strain was IMP-producer. In addition, one *Raoultella planticola* strain was tested to be KPC-producer. By colloidal gold immunochromatography, 59 out of 66 strains were positive for the detection of carbapenemase genes, including NDM (n = 37), KPC (n = 17), IMP (n = 4), and one expressed both NDM and OXA-48-like genes. Taking PCR as the gold standard, 5 of them were false positive, and the sensitivity and accuracy of colloidal gold immunochromatography were 100% and 92.4%, respectively.

### Treatment regimens

The definitive antimicrobial regimens were shown in Table 2. Overall, 28 cases received an antimicrobial treatment containing ceftazidime-avibactam (CAZ-AVI), of which 21 cases were combined with aztreonam (ATM) and 7 cases were not. The remaining 66 patients were treated with other active antibiotics (OAAs), including tigecycline, aminoglycoside, polymyxin, fosfomycin, fluoroquinolone and carbapenem. In the OAAs group, 25 patients received monotherapy and 41 patients received combination therapy. Tigecycline plus aminoglycoside with/without carbapenem was the most common combination therapy. Appropriate antibiotic therapy was started in 49 (52.1%) patients within 24 h of BSI onset, and in 83 (88.3%) patients within 48 h of BSI onset. Patients with a history of MDRO colonization/infection in the previous 3 months were more likely to receive active therapy within 24 h of infection (60.7% vs. 39.5%, *p* = 0.043).

**Table 2:** Antimicrobial treatments in patients with CRE BSI

Antimicrobial regimens	Total N (%)	30-day mortality N (%)	*P* value
**CAZ-AVI-containing regimen**	28 (29.8)	2/28 (7.1)	0.003
CAZ-AVI	3	0	
CAZ-AVI + ATM	7	0	
CAZ-AVI + ATM + tigecycline	10	0	
CAZ-AVI + ATM + aminoglycoside	2	0	
CAZ-AVI + ATM + polymyxin	2	1/2	
CAZ-AVI + tigecycline	4	1/4	
**OAAs regimen**	66 (70.2)	25/66 (37.9)	
Tigecycline-containing regimens	44 (66.7)	17/44 (38.6)	
Tigecycline + aminoglycoside	28	10/28	
± carbapenem			
Tigecycline + aminoglycoside	3	2/3	
+fluoroquinolone			
Tigecycline + fosfomycin	2	0	
Tigecycline + ATM	1	0	
Tigecycline ± carbapenem	10	5/10	
Polymyxin-containing regimens	10 (15.2)	4/10 (40)	
Polymyxin + tigecycline +	4	1/4	
aminoglycoside/ fluoroquinolone			
Polymyxin + tigecycline ± carbapenem	3	2/3	
Polymyxin + tigecycline + ATM	2	0	
Polymyxin alone	1	1/1	
Others	12 (18.2)	4/12 (33.3)	
Aminoglycoside ± carbapenem	9	4/9	
Fluoroquinolone + carbapenem	2	0	
Aminoglycoside + fluoroquinolone	1	0	
Monotherapy ^a^	25 (37.9)	13/25 (52.0)	
Combination therapy ^b^	41 (61.1)	12/41 (29.3)	0.065
**Appropriate therapy started within 24 h**			0.006
Yes	49 (52.1)	8/49 (16.3)	
No	45 (47.9)	19/45 (42.2)	
**Appropriate therapy started within 48 h**			
Yes	83 (88.3)	22/83 (26.5)	0.192
No	11 (11.7)	5/11 (45.5)	

CAZ-AVI, ceftazidime-avibactam; ATM, aztreonam; OAAs, other active antibiotics

^a^ only a single in vitro sensitive drug was used in the regimen

^b^ two or more active drugs was used in the regimen

### Outcomes

**Fig. 3:**
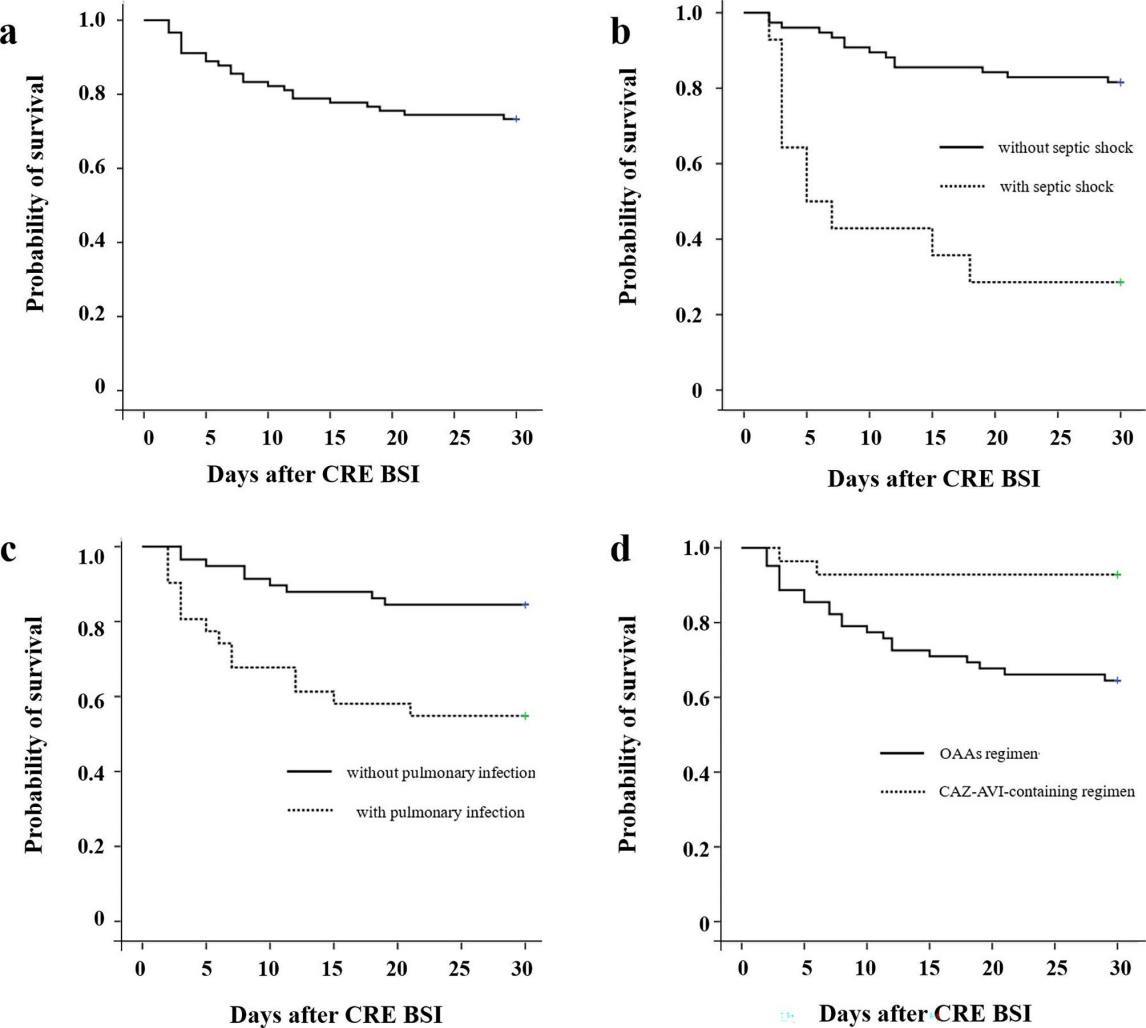
Kaplan-Meier curve of the 30-day survival probability of all patients with CRE BSI **(a)** patients with and without septic shock **(b)** patients with and without pulmonary infection **(c)** patients with ceftazidime-avibactam (CAZ-AVI) or other active antibiotics (OAAs) treatment **(d)**

The overall 30-day mortality rate was 28.7% (27/94), and the Kaplan-Meier survival curve was depicted in Fig. 3a. There was a median of 7 (range, 2–29) days from CRE BSI onset to death. Univariate analysis of risk factors for 30-day mortality was shown in Table 1. Patients with high-risk hematological diseases, prolonged neutropenia (≥ 14 days), septic shock and the PBS ≥ 2 were associated with increased mortality rate. In addition, co-infection with pneumonia was also risk factor for 30-day mortality, while appropriate empirical therapy administrated within 24 h of BSI onset was protective factors. We did not detect significant differences with regard to age, sex, Charlson Index, history of HSCT.

For patients treated with CAZ-AVI, the 30-day mortality rate was only 7.1% (2/28), which was significantly lower than those treated with OAAs (37.9%, 25/66, *p* = 0.003). In the OAAs group, compared with monotherapy, there was a trend towards decreased 30-day mortality in patients receiving combination therapy (29.3% vs. 52%, *p* = 0.065). In multivariate analysis (Table 3), the presence of septic shock at BSI onset (OR 10.526, 95% CI 1.376–76.923) (Fig. 3b) and pulmonary infection (OR 6.289, 95% CI 1.351–29.412) (Fig. 3c) were independently risk factors for 30-day mortality. Comparing different antimicrobial regimens, CAZ-AVI showed a significant survive benefit than OAAs (OR 0.068, 95% CI 0.007–0.651) (Fig. 3d).

**Table 3:** Multivariate analyses of risk factors for 30-day mortality in patients with CRE BSI

Risk factor	Multivariate analysis OR (95% CI)	*P* value
Underlying hematological disease	0.337 (0.048–2.367)	0.274
Length of neutropenia (≥ 14 days)	3.731 (0.355-40)	0.272
Pitt bacteremia score (≥ 2)	4.464 (0.902–22.222)	0.067
Septic shock	10.526 (1.376–76.923)	0.023
Pulmonary infection	6.289 (1.351–29.412)	0.019
Appropriate therapy started within 24 h	0.288 (0.066–1.255)	0.097
CAZ-AVI-containing regimen	0.068 (0.007–0.651)	0.020

OR, odds ratio; CI, confidence interval; CAZ-AVI, ceftazidime-avibactam

## Discussion

The increase in infections caused by CRE is a great challenge for patients with hematologic malignancies. Owing to immunocompromised status, prolonged hospitalizations, frequent antimicrobial use and neutropenia, those patients have a higher risk for CRE infection and treatment failure. The GITMO performed a retrospective study based on data from 52 stem cell transplant centers and demonstrated a CRE-related mortality rate of 64.4% in allogeneic HSCT recipients [6]. Our present study described the clinical outcomes and microbiological characteristics of 94 hematological patients with BSI due to CRE from 2012 to 2021. The result was encouraging, with 30-day mortality rate was 28.7% for all 94 patients, and was only 7.1% for 28 patients treated with CAZ-AVI.

Studies in the literature have indicated that timely and appropriate administration of empirical therapy is essential for managing CRE BSI [7–9]. Falcone M et al. evaluated risk factors for mortality in 102 patients with KPC-producing *K. pneumoniae* bacteremia and found median time to appropriate antibiotic therapy was shorter in patients who survived (8.5 h) versus those who died (48 h). Receipt of an in vitro active therapy within 24 h was independently associated with lower 30-day mortality (HR = 0.36, *p* = 0.0021) [9]. In the present study, we also observed a lower percentage of mortality among cases who received appropriate empirical therapy within 24 h of BSI episode (16.3% vs. 42.2%, *p* = 0.006). Further analysis, patients with MDRO colonization or infection in the previous 3 months tended more to receive active therapy within 24 h of infection. Therefore, close monitoring of CRE colonization is essential for initiating timely therapy once upon the onset of CRE infection.

Prior to the introduction of CAZ-AVI, the treatment of CRE infections was based on limited last-resort agents, such as tigecycline, polymyxins, fosfomycin and aminoglycosides. Though the optimal treatment for CRE BSI has not been well established, combination therapy with at least two active agents has been recommended in most published studies [10–12]. In addition, some studies suggested that combination therapy rather than monotherapy may be more beneficial for patients with septic shock or a high mortality score [13, 14]. A multicenter retrospective study investigated the effect of appropriate therapy on mortality of 437 patients with BSIs due to carbapenemase-producing Enterobacteriaceae. Though overall mortality was not different between those receiving combination therapy or monotherapy, lower mortality had been associated with combination therapy among patients with high-mortality-score stratum (48% vs. 62%, *p* = 0.020) [13]. Analyzing clinical outcomes of 66 patients treated with OAAs in our study, we also observed a trend towards decreased 30-day mortality in patients receiving combination therapy compared with monotherapy (29.3% vs. 52%, *p* = 0.065). Therefore, combination therapy is recommended for CRE BSI, especially for critically ill patients.

Ceftazidime-avibactam was the first new antibiotic approved by the US Food and Drug Administration (FDA) in 2015 for the treatment of CRE infections. Avibactam, a non-β-lactam β-lactamase inhibitor, has activity against Ambler class A and certain class D carbapenemases but not against class B metallo-lactamases [15]. Most studies published [8, 16–19] have demonstrated the survival benefit of CAZ-AVI in treatment of CRE infection, including higher clinical success, decreased mortality, and lower toxicity. Tumbarello M et al. [17] conducted the largest studies to date evaluating the efficacy of CAZ-AVI for KPC-*K. pneumoniae* infections. A total of 577 patients were included, and they received treatment with CAZ-AVI alone or with ≥ 1 other active antimicrobials. The all-cause mortality rate 30 days after infection onset was 25%, which was significantly lower than rates achieved with earlier non-CAZ-AVI-based drug regimens. Shields RK et al. [19] also indicated superiority of ceftazidime-avibactam to other treatment regimens against CRKP bacteremia. Moreover, nephrotoxicity was significantly less likely with ceftazidime-avibactam than with colistin- or aminoglycoside-containing regimens. Against MBLs, the combination of CAZ-AVI and ATM represents a promising treatment option. ATM remains hydrolytic activity against MBLs, however, it cannot be used alone due to frequent co-production of other enzymes (e.g., ESBLs, OXA-48) by MBL-producing Enterobacteriaceae [20]. Emerging data [21–23] has supported the combination of CAZ-AVI and ATM for MBL producers. A recent prospective study [21] evaluated the efficacy of CAZ-AVI plus ATM in patients with BSIs due to MBL-producing Enterobacteriaceae. Overall, 52 patients received a combination therapy of CAZ-AVI + ATM, whereas 50 were treated with OAAs. The 30-day mortality rate was significantly lower in the CAZ-AVI + ATM group than in the OAAs group (19.2% vs. 44%, *P* = 0.007). In our study, a total of 28 patients received an antimicrobial treatment containing CAZ-AVI. Corroborating previous studies, we also observed a remarkable decreased mortality for patients treated with CAZ-AVI than with OAAs (7.1% vs. 37.9%, *p* = 0.003). In multivariate analysis, the administration of CAZ-AVI was the independent predictor for favorable outcome. With regard to the obvious survival benefit, we considered CAZ-AVI as the preferred treatment option for CRE BSI.

As the distribution of carbapenemases varies in nations, regions and even centers, knowledge of the prevalence and molecular characteristics of CRE is of vital importance. KPC-producing Enterobacteriaceae are widespread in the United States, Latin America, Italy and Greece [24]. NDM-producing Enterobacteriaceae are mainly detected in Indian subcontinent [25], while OXA-48-producing Enterobacteriaceae are endemic in Turkey [26]. In China, *bla*KPC and *bla*NDM are the most common carbapenemase genes among CRE strains. Data from a longitudinal large-scale CRE study in China (2012–2016) proved that KPC and NDM are the major carbapenemases produced by CRE, while KPC was predominant in *K. pneumoniae* (77%), NDM was predominant in *E. coli* (75%) and *E. cloacae* (53%) [27]. Another study [28] collected 935 non-duplicate CRE strains from 36 hospitals across China, and reached similar conclusions. In our study, NDM (66.7%, 36/54) was the most common carbapenemase gene, followed by KPC (29.6%, 16/54). Different from other centers in China, all 25 carbapenemase-producing *E. coli* in our study were identified NDM gene. Besides, NDM also accounted for a large proportion (37.5%, 9/24) in carbapenemase-producing *K. pneumoniae*. Considering the predominance of NDM gene in our center, the majority (75%, 21/28) of patients in CAZ-AVI group received the combination with ATM, which contributed to the decreased mortality and higher clinical success. Therefore, detection of Carbapenemase genes was crucial as it could guide the rational choices of antibiotics.

The present study had several limitations. First, it was a single-center retrospective study, designed exclusively for hematological patients. The results from our study may not be representative of the experience at other centers. Second, considering the heterogeneous treatment regimens in the study, we did not perform further comparison for mortality between different antibiotics or combinations. Third, though we observed a distinct advantage of CAZ-AVI therapy for CRE BSI, the number of patients in the CAZ-AVI group were relatively small. More large-scale studies are warranted.

In conclusion, this study indicates the severity and poor prognosis of CRE BSI in patients with hematologic malignancies. The initiation of appropriate empirical therapy within 24 h of BSI onset is crucial for managing CRE BSI. We confirm the superiority of CAZ-AVI to other treatment regimens against CRE BSI. Detailed knowledge of the prevalence trends and distribution of carbapenemase-producers is quite essential. Unlike previous reports in China, we found a larger proportion of *bla*NDM in carbapenemase-producing Enterobacteriaceae in our center. Taking this into account, we recommend the combination of CAZ-AVI with ATM when choose CAZ-AVI-containing regimen.

## Data Availability

Not applicable.
